# Laryngeal Phthisis

**Published:** 1882-04

**Authors:** J. D. Arnold

**Affiliations:** Baltimore, Lately Principal Clinical Assistant, Central Throat and Ear Hospital, London, England


*FOR CONTENTS OF THIS NUMBER SEE PAGE 288.*

*THE*

### Independent Practitioner.

VOL. III.——*April, 1882.*——NO. 4.

### ORIGINAL COMMUNICATIONS.

*We are not responsible for the opinions expressed by contributors.*

ARTICLE I.

### LARYNGEAL PHTHISIS.

BY J. D. ARNOLD, M.D., BALTIMORE,
Lately Principal Clinical Assistant, Central Throat and Ear Hospital, London, England.

The laryngoscopic appearances in throat consumption have of late years been so closely studied and so accurately noted that the diagnosis of the disease with the aid of the mirror alone has been made possible in the great majority of cases, and although it must sometimes happen that a differentiation between syphilis and tuberculosis of the larynx becomes difficult, few laryngoscopists of the present day often have occasion to put a patient upon a tentative anti-specific treatment to decide this point, as was advised by Tuerk and Semeleder. The clinical value of the fact becomes patent, when we remember that, although as yet no case of undoubted primary laryngeal tuberculosis has been observed, in many instances this process can be recognized in the throat with the laryngoscope before its presence can be detected in the lung with the stethoscope ; and, moreover, if we may indulge the hope that medicine will at some time discover a means of arresting this fell disease, it is certain that its early recognition must be an important factor in the employment of such means.

The tuberculous larynx rarely comes under the physician's observation in its incipiency, because until the development of ulceration so few symptoms arise that attest the presence of disease in the throat. In all other processes which have their seat in the mucous membrane of the larynx, and which result in the breaking down of tissue and loss of substance, the ulcer is preceded by an acute inflammation accompanied by the usual phenomena—increased vascularity, redness, swelling, tension, and painfulness; but the tuberculous ulcer is not the outcome of an active inflammation in its classical sense, and the conditions preceding its formation occasion the patient little distress and leave the functions of the parts almost unimpaired. Of course it is possible that in a tuberculous individual an acute laryngitis may occur which is non-tuberculous—that is, which does not arise from the presence of tubercle in the larynx—and such a laryngitis offers little to distinguish it from other inflammations in this region, except that it is less amenable to treatment.

The onset of the phthisical process in the larynx is often evidenced by an extreme anæmia of the mucous membrane, in decided contrast to the angry hyperæmia of acute catarrh, or the deep red color of the specific inflammation which always precedes the syphilitic sore. At some point upon this bleached surface will soon arise a small circumscribed tumescence which slowly broadens and finally breaks down into an irregular ulcer. More frequently the mucous membrane presents, in the first stage of the disease, an appearance closely resembling a chronic catarrh—with this difference, that all the structures in the larynx look thickened and give evidence of deep infiltration long before any point of ulceration can be seen. The loose disposition of the mucosa and submucosa over the arytenoids and ary-epiglottic folds offer less resistance to infiltration than that of the contiguous parts, and the œdematous appearance which those structures present in the laryngoscopic image is almost pathognomonic of throat consumption. Up to this time the epithelium itself has undergone no change, showing that the process originates within and extends toward, not from, the pale mucous surfaces. Quite limited districts, such as the anterior commissure of the cords, the inter-arytenoid space, or the margin of the epiglottis, may be for a long while the only parts attacked, but as a rule several of the different regions in the larynx are simultaneously infiltrated, lose their epithelium in patches, and expose to view pale, unhealthy granulations that slowly spread and give to the parts that mousenibbled appearance so characteristic of this disease. These granulations bear some resemblance to the hyperplasiæ which often form at the edges of syphilitic ulceration, but, unlike the latter, they are not overtures to repair, and evince no tendency to the formation of cicatrices.

The tuberculous ulcer is usually shallow, showing a disposition to spread laterally rather than in depth, and it is only in exceptionally severe cases that muscle and cartilage become implicated. Its surface has a peculiar grayish white color, and does not present those points of softening which characterize the sloughing sore of syphilis. What is most distinctive of the tuberculous process is the absence of all tendency toward repair, whereas in the ulcerative throat of syphilis—and it is only with syphilis that tuberculosis in this region is apt to be confounded—there may always be detected in those structures first attacked the growth of thin bands of white fibrous tissue, the earliest evidence of cicatrization. It is not unusual to see in the same throat the ventricular bands covered with deep sloughing sores, and cicatricial webs upon the cords or epiglottis—the process of repair progressing side by side with the process of disintegration, and this even in the absence of constitutional treatment; for syphilis, as far as its separate secondary manifestations are concerned, is a self-limiting disease. A condition resembling œdema usually is present on the edges of the tuberculous ulcer, especially when its seat is upon the arytenoids or epiglottis. That this is not a simple serous effusion is proved by its slow establishment and its absolute indifference to such measures as are potent in the reduction of ordinary watery infiltration. The arytenoids, when so affected, are firm, unyielding, and opaque, and lack that opaline, semi-translucent appearance which characterizes them when truly œdematous. I had once the good fortune, or rather bad fortune, to see the arytenoids scarified for the relief of such a condition, with the result that to the chronic plastic infiltration was superadded an acute, serous œdema which necessitated the performance of tracheotomy three hours after the scarification.

The favorite seat of the tuberculous ulcer is the vocal cords, and this is no doubt due to the comparatively greater amount of work which they have to perform in phonation; because, *a priori*, the cords from their structure should be better able to resist infiltration and disintegration than any other part of the larynx. After the cords, the inner surface of the inter-arytenoid space is the region most frequently the point of ulceration, and when, as it often happens, this is the only part attacked, it becomes quite difficult to determine the character of the ulceration.

from its appearance in the mirror. One sees merely small grayish or pale red elevations between the arytenoids which may readily be mistaken for papillamatous growths ; the experienced eye, however, recognizes in them the fringed edges of an ulcer inside the cavern of the larynx, whose face and surface can only be brought into view by placing the patient in the position for tracheoscopy. It is almost pathognomic of the phthisical throat that it is constantly deluged with frothy, mucous secretion, which is often, from its persistence, a hindrance to proper inspection of the parts.

The symptoms preceding the stage of ulceration are, as above stated, very unobtrusive, but when this has once set in, there arises progressive aphonia, due either to loss of substance in the cords, or to infiltration of the constrictors, or to the mechanical impediment offered to the approach of the vocal bands by the pyriform swelling of the arytenoids. Deglutition becomes painful because of the pressure exerted upon the sore parts by the muscles of the larynx, which contract that organ during the act of swallowing. A short, distressing, ineffectual cough is always present, but during the intervals between the paroxysms, no pain is experienced unless there exist necrosis of the cartilages from extension of the ulceration. Of course all those broad, general symptoms will exist which are to be referred to the pulmonary trouble that is always primary to the affection of the larynx.

The anatomical character of the ulceration in laryngeal phthisis has long been a fruitful theme of dispute among pathologists, and upon no other subject have the dicta of authorities been so radically different. Wunderlich and Louis declared that they never in a single case found tubercle in the larynx ; Waldenberg and Rindfleisch allow that tubercle may be deposited in the larynx and there by retrograde metamorphosis give rise to destructive ulceration, but believe that this is true only in a very small minority of cases ; while Andral, Rokitanski, and Virchow state that ulceration in the larynx in phthisical individuals nearly always owes its origin to the breaking down of tuberculous masses. When so accurate an observer as Rindfleisch asserts positively that tubercle is rarely, if ever, found in the larynx—and Virchow recommends this organ as just the region above all others for the histological study of true tubercle—it is reasonable to believe that these pathologists must differ just so widely in their definition of the term *tubercle* as their conclusions are different.

Since Laennec, who first observed that miliary tubercles agglomerate and undergo caseous degeneration, taught that whenever caseous metamorphosis takes place it must have such an origin, great confusion has existed as to what kind of structure actually constitutes tubercle ; and as an effect of his teaching there arose a disposition to call everything caseous-tuberculous, until finally the term miliary tubercle came almost into disuse. Virchow, whose name is so inseparably connected with modern pathology, brought order again into this chaos by showing that cheesy degeneration is not peculiar to any particular species of pathological tissue, but that under certain conditions of nutrition, pus, cancerous tissue, typhoid infiltration, and the like, could undergo the same change. He concluded, therefore, that the product of such change must not be identified with a new growth which makes its appearance in the shape of minute tumors and for which the term tubercle should properly be retained.

That tuberculosis of the larynx is always a secondary affection seems to have been firmly established by the valuable researches of Heinze and Eppinger ; and the belief that ulceration in the larynx was directly caused by contact of secretion from the phthisical lung has been entirely abandoned, since it is certain that the lymph and sometimes the blood vessels are the channels of tuberculous dissemination.

Heinze, in an exhaustive monograph upon "Throat Consumption," finds that among 4486 consecutive post-mortems made at the Pathological Institute of Leipsig, in 1226 cases pulmonary phthisis was the cause of death ; and of these 51.3 per cent had ulceration of the larynx. He further found—and this is of special interest—that ulceration of the larynx was never present in tuberculosis of other organs, when the lungs were intact. As to the influence of age and sex his statistics agree in general with those of Willig, Ziemssen, and Mackenzie. Concerning the character of the laryngeal ulcer, his materials for investigation were 50 patients from among those who died with pulmonary consumption during the year 1876 at the St. Jacob's Hospital, and the only basis for a choice of cases was that the throat should be in some way abnormal. Of this number 49 presented ulceration in the larynx, and 1 an intense catarrh, but no ulceration. Among these the ulceration was tuberculous in 83 per cent, and in 17 per cent non-tuberculous. Heinze, however, discovered that whenever tubercle could not be found in the laryngeal ulcer the loss of substance amounted only to an erosion of the mucous membrane and agreed in character with the aphthous, or, as Virchow has called it, the lenticular ulcer, which has also been found in the throat affections of the various exanthemata. After a searching analysis of his careful classical and histological observations, Heinze deduces, firstly : that laryngeal phthisis always owes its origin to tuberculosis of the laryngeal mucous membrane ; secondly, that tuberculosis of the larynx is never primary, neither in the sense that tubercle can develop in the larynx before the lungs are affected, nor in the sense that the larynx may take part in a general tuberculosis when the lungs remain intact ; and thirdly, that tuberculous ulcers never heal, understanding by the healing of an ulceration the formation of cicatrix.

The chapter of therapeutics in tuberculosis is notoriously short and unsatisfactory, and outside of a greater disposition than formerly on the part of clinicians to use medicaments in the form of spray and vapor, the painstaking histological research which this disease has commanded for the past decade has wrought no great change in its treatment. Such measures as look toward the establishment of an improved physical tone in the patient—whether they be climatic, dietetic, or purely medicinal—must demand the most careful consideration and employment in the absence of any special treatment that can reasonably be expected to arrest or retard this malady. To such a general symptomatic and expectant method there is little to be added in the management of the local affection in the larynx. When one has used morphia either in vapor, spray, or insufflation to ameliorate the distress that accompanies cough and deglutition ; when one has exhausted the ice-bag and dilator in their use as surrogates for tracheotomy when dyspnœa from stenosis threatens life, one has done all that is useful, perhaps all that is safe, in laryngeal phthisis. Special remedies have never found favor or produced expected results in other than special hands, and that a certain quantity of milk per diem will cure the phthisical patients of him who first proposed the milk cure is just as natural as that a decoction of flint stones should annihilate tuberculosis in the patients of the discoverer of this powerful medicament. In the long list of drugs, from oleum pini sylvestris to arsenic, that have been proposed and used in this disease, no one has really made good its claims to the slightest curative value in tuberculosis. A gloomy picture, surely, but unfortunately the investigations of pathologists and the experience of clinicians have as yet supplied no brighter tint to lighten up the sombre coloring.

